# Real-life experience with benralizumab during 6 months

**DOI:** 10.1186/s12890-020-01220-9

**Published:** 2020-06-29

**Authors:** A. Padilla-Galo, RCh Levy-Abitbol, C. Olveira, B. Valencia Azcona, M. Pérez Morales, F. Rivas-Ruiz, B. Tortajada-Goitia, I. Moya-Carmona, A. Levy-Naon

**Affiliations:** 1 Pneumology Unit, 4th floor. Agencia Sanitaria Costa del Sol. Carretera Nacional 340, Km 187, 29603, Marbella, Málaga, Spain; 2 Yeshiva University, New York, USA; 3 Pneumology Department, IBIMA (Institute for Biomedical Research of Málaga), Regional University Hospital of Málaga/ University of Málaga, Málaga, Spain; 4 Avenida Carlos Haya, 29010 Málaga, Spain; 5 Research Unit, Red de Investigación en Servicios de Salud en Enfermedades Crónicas, REDISSEC (Spanish healthcare network for chronic diseases), Agencia Sanitaria Costa del Sol. Carretera Nacional 340, Km 187, 29603, Marbella, Málaga, Spain; 6 Pharmacy and Nutrition Service, Agencia Sanitaria Costa del Sol. Carretera Nacional 340, Km 187, 29603 Marbella, Málaga Spain; 7 Pharmacy and Nutrition Service, Hospital Universitario Virgen de la Victoria, Campus de Teatinos s/n, 29010 Málaga, Spain; 8 Pneumology Department, Hospital Universitario Virgen de la Victoria, Campus de Teatinos s/n, 29010 Málaga, Spain

**Keywords:** Asthma, Benralizumab, Eosinophils, Biologics, Severe asthma, Eosinophilic asthma, Real-life

## Abstract

**Background:**

Benralizumab is a monoclonal antibody that binds to the human interleukin-5 (IL-5) receptor (IL-5R), thereby preventing IL-5 from binding to its receptor and inhibiting differentiation and maturation of eosinophils in the bone marrow. Because of its recent marketing approval, sufficient real-life evidence is lacking to confirm the efficacy and safety data from clinical trials. The purpose of this study was to evaluate the efficacy and safety of benralizumab for the treatment of severe refractory eosinophilic asthma in a real-world cohort of patients.

**Methods:**

This was a cross-sectional multicentre study of consecutive patients with severe refractory eosinophilic asthma who received treatment with benralizumab during at least 6 months. Patient follow-up was performed in specialised severe asthma units.

**Results:**

A total of 42 patients were enrolled and treated with benralizumab. Asthma control, as measured by the asthma control test (ACT), improved in all patients both at 3 months of treatment compared with baseline (13.9 ± 4 vs 20.1 ± 3.7, *p* < 0.001) and at 6 months of treatment compared with the results obtained at 3 months (20.1 ± 3.7 vs 21 ± 2.7, *p* = 0.037). Similarly, the number of emergency department visits decreased both at 3 months compared with baseline (1 [IR:0.7] vs 0 [IR:0.75], *p* < 0.001) and at 6 months compared with the results at 3 months (0 [IR:0.75] vs 0 [IR:0], *p* = 0.012). Reductions in the number of oral corticosteroid cycles, percentage of corticosteroid-dependent patients, and mean daily dose of oral or inhaled corticosteroid were also evidenced. Finally, mean lung function improvement was 291 mL (*p* < 0.001), and FEV1% improved both at 3 months compared with baseline (64.4 ± 9.3 vs 73.1 ± 9.1, *p* < 0.001) and at 6 months compared to 3 months (73.1 ± 9.1 vs 76.1 ± 12, *p* = 0.002). Side effects were mild and did not lead to treatment discontinuation.

**Conclusions:**

This study confirms the efficacy and safety of benralizumab in a real-life setting with improved asthma control and lung function, and a reduced oral and inhaled corticosteroid use as well as fewer emergency department visits. In addition to a rapid initial improvement, it appears that patients continue to improve during the first 6 months of treatment.

## Background

Asthma is a heterogeneous condition characterised by chronic inflammation of the pulmonary airways, [1] with an estimated 300 million people currently affected worldwide [2] and an increasing prevalence. Severe asthma is defined, following a confirmed diagnosis of asthma and appropriate treatment of comorbidities, as asthma requiring high-dose inhaled corticosteroids (ICS) plus a second controller and/or systemic corticosteroids to prevent it from becoming “uncontrolled” or remaining “uncontrolled” despite this therapy (refractory) [1, 3]. Its prevalence is estimated at 5–10% of the total asthma population [4]. In addition, several studies have shown that, despite the availability of effective treatments such as ICS, long-acting β2-agonists (LABA), leukotriene modifiers, and tiotropium, over 50% of asthma patients are assessed as not well-controlled in standard clinical practice [5, 6] and many require further therapies such as oral corticosteroids (OCS) and biologics [7]. Over 50% of deaths caused by asthma are reported in patients with a history of severe asthma [8], and this severe condition is associated with increased healthcare costs and morbidity and mortality [9, 10]. Furthermore, from the patient’s point of view, severe asthma can be an incapacitating disease as well as a threat to identity and life roles [11], and severe asthma patients using OCS are at higher risk of complications such as diabetes or hypertension. These OCS-related comorbidities increase the burden of disease for patients and healthcare providers [12, 13].

In the recently updated Global Initiative for Asthma (GINA) guidelines, the use of biologics before using maintenance OCS is recommended in step 5. In Europe, as of March 2020, there were four marketed biologics for asthma: omalizumab (Xolair®, Novartis Pharma GmbH), an anti-IgE monoclonal antibody that selectively binds to human IgE and prevents its binding to high-affinity IgE receptors [14], indicated in Europe as an add-on therapy to improve asthma control in patients with severe persistent allergic asthma; mepolizumab (Nucala®, GlaxoSmithKline) [15] and reslizumab (Cinqaero®, Teva) [16], both targeting interleukin-5 (IL-5) and indicated in severe eosinophilic asthma; and benralizumab (Fasenra®, AstraZeneca), the most recently marketed monoclonal antibody to date. The latter binds to the human IL-5 receptor (IL-5R) through its Fab domain, thereby preventing IL-5 from binding to its receptor and inhibiting differentiation and maturation of eosinophils in the bone marrow. In addition, this antibody has the ability to bind through its afucosylated Fc domain to the RIIIa region of the Fc receptor on natural killer cells, macrophages, and neutrophils, thereby enhancing antibody-dependent cell-mediated cytotoxicity (ADCC) of both blood eosinophils and tissue-resident eosinophils [17, 18].

Given that monoclonal antibodies are expensive, clear indications should be established to optimise access to these therapies and ensure maximum efficiency [19, 20]. Thus, prior to initiating treatment with a biologic agent, patients with severe asthma should be classified into phenotypes. Among the various asthma phenotypes/endotypes, late-onset eosinophilic asthma is, together with allergic asthma, one of the best defined phenotypes and the most common clinical phenotype seen in the specialised severe asthma units of Pneumology departments in Spain [21]. The definitive diagnosis of eosinophilic asthma is based on an appropriate medical record and evidence of eosinophilia in bronchial biopsies or induced sputum, which can also be estimated with a reasonable accuracy in peripheral blood. Eosinophilic inflammation occurs in more than 50% of patients with allergic and non-allergic asthma, and elevated eosinophil counts in both peripheral blood and airways are associated with recurrent exacerbations of the disease and severe airflow limitation [22]. Eosinophilic asthma is driven by type-2 inflammatory mechanisms that are dependent on the activity of T helper-2 lymphocytes and group 2 innate lymphoid cells [23, 24]. Among the wide range of pro-inflammatory mediators released by these cells, IL-5 is the key cytokine responsible for most of the functions of eosinophils, including their maturation in the bone marrow, activation, chemotaxis, survival, and proliferation [25]. In addition, type-2 asthma generally responds well to ICS [26] which are potent inducers of eosinophil apoptosis [27]. However, some patients with eosinophilic asthma respond poorly to ICS and even to OCS [28]. Therefore, the eosinophil pathway and IL-5 pathway are appropriate therapeutic targets in patients with corticosteroid-refractory severe eosinophilic asthma. Benralizumab, as an interleukin-5 receptor alpha–directed cytolytic humanised IgG1k monoclonal antibody, induces direct, rapid, and nearly complete depletion of eosinophils via enhanced ADCC [29].

In the phase 3 clinical trials conducted (SIROCCO [30], CALIMA [31], and ZONDA [32]), benralizumab reduced the annual rate of severe asthma exacerbations and the use of OCS, and improved symptom control and lung function determined by the forced expiratory volume in 1 s (FEV1). Additionally, the BORA study [33] has shown its long-term efficacy and safety. However, because of the recent marketing approval of benralizumab, few real-life data are available to date. Therefore, the aim of this study was to assess the efficacy of benralizumab in real life based on the assessment of symptom control, emergency department visits, use of oral and inhaled corticosteroids, lung function, and safety at 6 months of treatment.

## Methods

### Study population

This multicentre study included 42 consecutive patients with severe refractory eosinophilic asthma who received treatment with benralizumab for at least 6 months at the Asthma Units of Hospital Costa del Sol (Marbella, Spain) and Hospital Virgen de la Victoria (Málaga, Spain), from January 2019 to November 2019. All patients were diagnosed by objective tests (FEV_1_ reversibility ≥12%, positive results to methacholine, or FEV_1_ variability ≥20%).

We used a standardised protocol to try to improve these patients’ asthma control. This consisted of ensuring adherence to both therapy and appropriate inhaler use, providing health education, adjusting treatment, and ruling out comorbidities [34–36].

Benralizumab treatment initiation criteria were as follows:

- 18 year-old patient or older with severe refractory asthma [3];
- GINA guidelines step 5 [1];
- 2 or more exacerbations during the previous year with use of OCS despite receiving appropriate treatment for the degree of severity or corticosteroid dependence;
- Presence of eosinophilic inflammation: eosinophil count ≥300 cells/μL in peripheral blood during the previous 12 months or ≥ 150 cells/μL in case of corticosteroid dependence.

All patients were treated with benralizumab for at least 6 months and were included in the analysis.

Patients previously treated with another biologic agent but who had failed to respond, based on the physician’s judgement, were included. The following criteria for lack of response to a prior biologic treatment were applied:

- Continued use of maintenance OCS despite receiving biologic therapy for at least 12 months, or.
- Less than a 50% reduction in exacerbations after at least 12 months of biologic therapy.

Following treatment initiation with benralizumab, at least two visits were performed: one at 3 months of treatment and one at 6 months of treatment.

Written informed consent was obtained from all participants. The study was reviewed by the Spanish Medicines and Health Products Agency and approved by the ethics committee *Comité de ética provincial de Málaga*.

### Clinical, analytical, and lung function variables

A database was compiled from complete medical records, with data from diagnosis to enrolment in the study. A standardised protocol was applied for the prospective collection of sociodemographic data (sex, age), clinical profile (age at diagnosis of asthma, atopy, presence of nasal polyps), exacerbations, use of corticosteroid therapy, and basic blood test. Dyspnoea was evaluated by means of the modified Medical Research Council Scale for Dyspnoea [37], and we divided patients into two stage groups, 0–2 and 3–4, according to their degree of dyspnoea. We used the asthma control test (ACT) to evaluate the degree of asthma control in the 4 weeks prior to the clinical interview. The ACT [38] is a self-administered tool that is easy for patients to complete. It includes four symptom-relief questions plus a patient’s self-assessment of asthma control [1] in the last 4 weeks, with scores ranging from 5 (poor control) to 25 (complete control), and has been validated in Spanish [39]. Nasal polyposis was diagnosed by an otorhinolaryngologist by direct visualization of the polyps with endoscopic examination. Patients were considered as atopic when they had positive allergic prick tests or positive specific IgE to the most prevalent pneumo-allergens in our area (house dust mites, olive tree and grass pollen among other types of pollen, fungi, and animal epithelium such as that of dogs and cats), provided that these positive findings also had clinical relevance. Corticosteroid dependence was defined as the daily use of OCS during at least 6 months.

All patients were trained to identify exacerbation symptoms. They were also asked to record detailed information about their condition and their prescriptions (systemic steroids). This information was verified in their medical records.

Fractional exhaled nitric oxide (FeNO) was measured with a conventional chemiluminescence analyser (NIOX, Aerocrine AB, Sweden) using the online standardised single-breath technique, and was followed by the performance of a spirometry. Both procedures conformed to international guidelines [40, 41].

All the variables were measured during the baseline visit and at 3 and 6 months of treatment.

### Statistical analysis

All the data were analysed using the R statistics system [42]. A descriptive analysis was performed using measures of central tendency, position, and dispersion for quantitative variables, and frequency distribution for qualitative variables. To assess changes at 3 and 6 months compared with baseline, Student’s t test for paired samples was used (Wilcoxon rank test for non-normal distributions) and McNemar test was used for qualitative variables. Statistical significance was set at *p* < 0.05.

## Results

### Clinical characteristics

A total of 42 patients with severe refractory eosinophilic asthma who received benralizumab treatment during at least 6 months were enrolled. Clinical characteristics of the study population are shown in Table 1.

**Table 1:** Baseline patient characteristics

Parameter	*n* = 42
Age, years (m ± sd)	53.6 ± 11
Women, n (%)	33 (78.6)
BMI (m ± sd)	28.6 ± 6
Age at diagnosis, years (m ± sd)	28.9 ± 12.6
Dyspnoea	
Degree 0–2, n (%)	20 (47.6)
Degree 3–4, n (%)	22 (52.4)
Atopy, n (%)	14 (33)
Corticosteroid-dependent, n (%)	17 (40.5)
Nasal polyps, n (%)	12 (28.6)
AERD, n (%)	6 (14.3)
ACT (m ± sd)	13.9 ± 4.1
ED visits in the previous year (m ± sd)	4.1 ± 2.6
Cycles of OCS in the previous year, (m ± sd)	5.9 ± 3.3
Oral prednisone (or equivalent) dose, mg/day (m ± sd)	19.6 ± 9
Inhaled budesonide (or equivalent) dose, μg/day (m ± sd)	956 ± 475
Post-BD FEV_1_, mL (m ± sd)	1429.4 ± 475
Post-BD FEV_1_, % (m ± sd)	64.4 ± 9.3
FeNO, ppb (m ± sd)	61.5 ± 23.9
Blood eosinophil count, cells/μL (m ± sd)	757.2 ± 278
IgE, IU/mL (m ± sd)	228.6 ± 403
Prior treatment with a biologic agent, n (%)	22 (52.4)
Prior omalizumab, n (%)	15 (35.7)
Prior mepolizumab, n (%)	5 (11.9)
Prior omalizumab + mepolizumab, n (%)	2 (4.8)
Duration of prior biologic therapy, months (m ± sd)	21.14 ± 23.8
Duration of prior omalizumab therapy, months (m ± sd)	26.2 ± 27.5
Duration of prior mepolizumab therapy, months (m ± sd)	9.8 ± 3.5

Abbreviations: *ACT* Asthma Control Test, *AERD* Aspirin-exacerbated respiratory disease, *BD* Bronchodilator, *BMI* Body mass index, *ED* Emergency department, *FeNO* Fractional exhaled nitric oxide, *FEV*_*1*_ Forced expiratory volume in 1 s, *m* Mean, *ppb* Parts per billion, *OCS* Oral corticosteroids, *sd* Standard deviation

### Parameters assessed

Clinical, functional, and laboratory data at baseline and at 3 and 6 months of treatment as well as the comparison between values at baseline and at 6 months are presented in Table 2.

**Table 2:** Clinical, functional, and laboratory data at baseline and at 3 and 6 months of treatment

Variables	BaselineMean (SD)	3 months Mean (SD)	6 months Mean (SD)	p*
ACT	13.9 (4.1)	20.1 (3.7)	21 (2.7)	**< 0.001**
Patients with controlled asthma (ACT≥20)	2 (4.8)	24 (57.1)	34 (81)	**< 0.001**
No. of ED visits per quarter, median (interquartile range)	1 (IR:0.7)	0 (IR:0.75)	0 (IR:0)	**< 0.001**
Corticosteroid-dependent, n (%)	16 (40%)	15 (35.7%)	8 (19%)	**0.008**
Inhaled budesonide (or equivalent) dose, μg/day	956 (475)	802 (415)	714 (356)	**0.001**
Oral prednisone dose, mg/day	19.6 (9)	7.5 (8.6)	5 (7.9)	**0.007**
Cycles of OCS per quarter, median (interquartile range)	1.5 (IR:1.25)	0 (IR:1)	0 (IR:0)	**< 0.001**
FEV_1_ mL	1429.4 (475)	1680 (481)	1721 (610)	**< 0.001**
FEV_1_%	64.3 (9.3)	73.1 (9.1)	76 (12)	**< 0.001**
FeNO	61.5 (23.9)	30.6 (14.6)	27.8 (14.9)	**< 0.001**
Blood eosinophil count, cells/μL	757.2 (278)	18.9 (18)	15.2 (13.6)	**< 0.001**

* Comparison between data at baseline and at 6 months

Abbreviations: *ACT* Asthma Control Test, *BD* Bronchodilator, *BMI* Body mass index, *ED* Emergency department, *FeNO* Fractional exhaled nitric oxide, *FEV1* Forced expiratory volume in 1 s, *OCS* Oral corticosteroids, *SD* Standard deviation

**Fig. 1:**
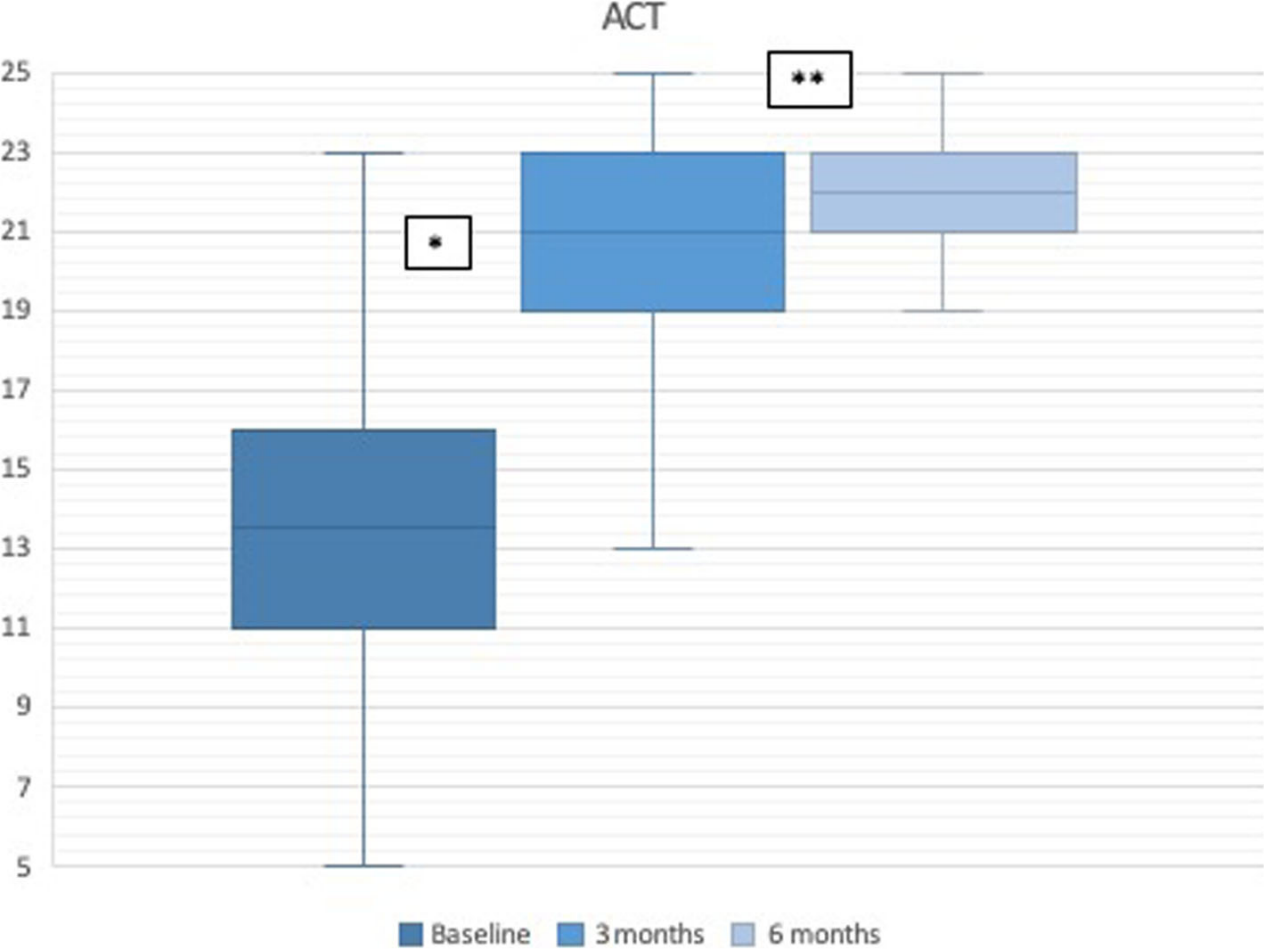
Asthma Control Test scores. * *p* < 0.001 (baseline vs 3 months); ** *p* = 0.037 (3 months vs 6 months)

As illustrated in Fig. 1, patients showed improved asthma control based on the ACT scores at 3 months of treatment compared with baseline (13.9 ± 4 vs 20.1 ± 3.7, *p* < 0.001), and continued to improve at 6 months compared with the values obtained at 3 months (20.1 ± 3.7 vs 21 ± 2.7, *p* = 0.037). Less than 5% of patients showed adequate asthma control before initiating benralizumab, whereas 81% of patients had achieved asthma control at 6 months of treatment (Table 2).

**Fig. 2:**
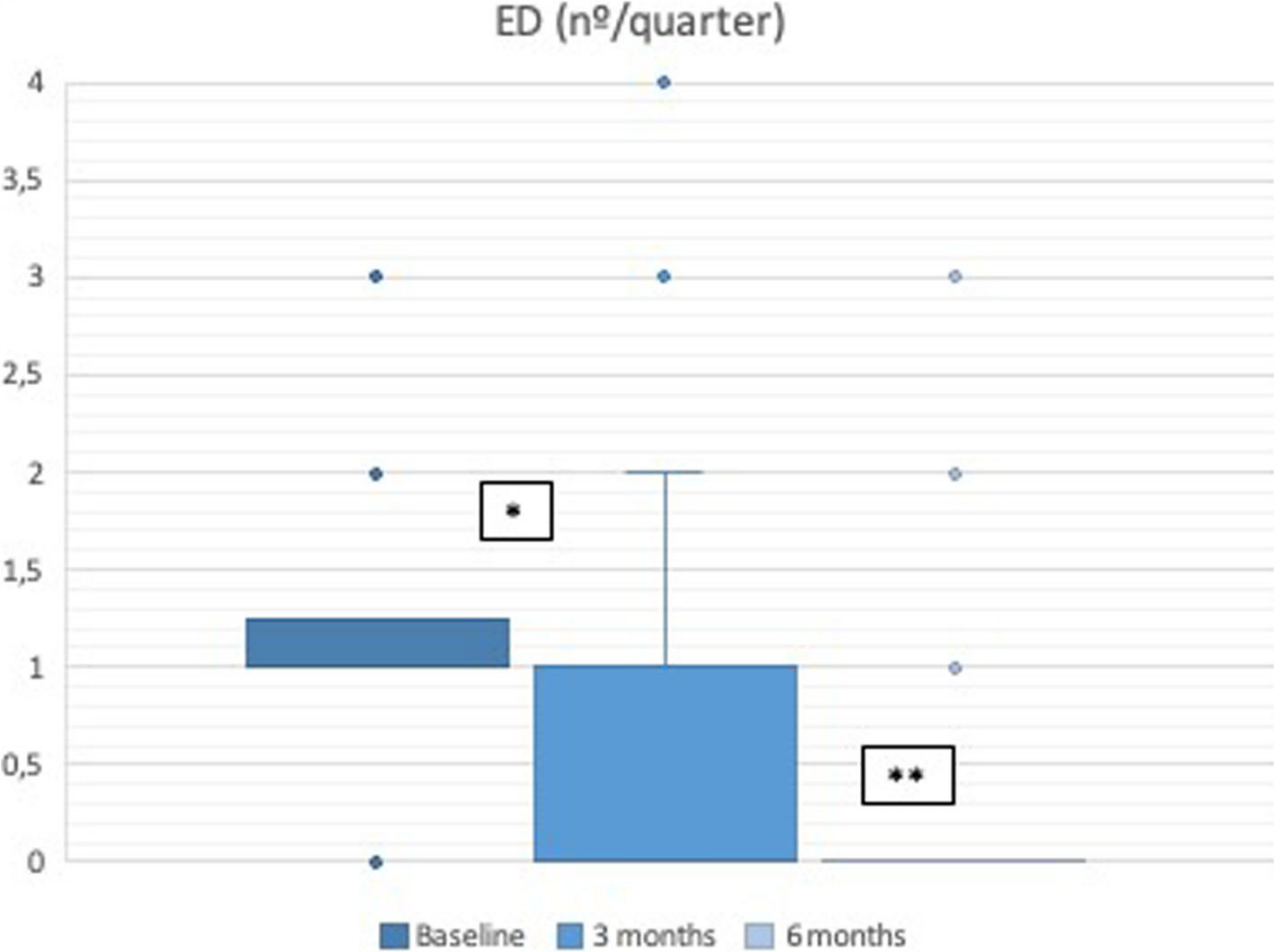
Emergency department visits. * *p* < 0.001 (baseline vs 3 months); ** *p* = 0.012 (3 months vs 6 months)

We compared the estimated number of emergency department visits per quarter prior to starting benralizumab and after 3 and 6 months of treatment with benralizumab (Fig. 2). A reduction in the number of emergency department visits both at 3 months compared with baseline (1 [IR:0.7] vs 0 [IR:0.75], *p* < 0.001) and at 6 months compared with 3 months (0 [IR:0.75] vs 0 [IR:0], *p* = 0.012) was observed.

**Fig. 3:**
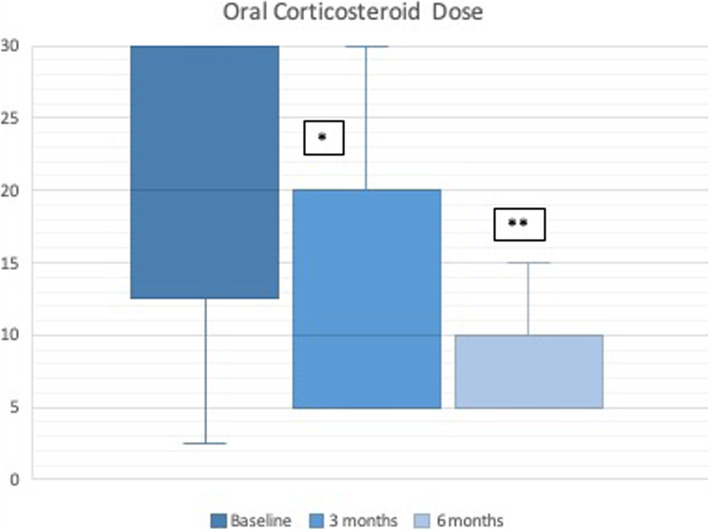
Daily dose of prednisone (mg). * *p* < 0.001 (baseline vs 3 months); ** *p* = 0.020 (3 months vs 6 months)

With regard to OCS use (Fig. 3), the dose of prednisone in mg (or equivalent) at 3 months of benralizumab treatment compared with baseline decreased (19.6 ± 9 vs 7.5 ± 8.6, *p* = 0.001) and continued to do so up to the 6 months of treatment, reaching a mean dose of 5 mg ± 7.9 of prednisone/day, with a statistically significant decrease compared with the dose at 3 months (*p* = 0.020).

We compared the estimated number of OCS cycles per quarter prior to starting benralizumab and at 3 and 6 months of benralizumab treatment to look for changes in OCS cycle patterns. We found that the number of OCS cycles decreased both at 3 months (1.5 [IR:1.25] vs 0 [IR:1], *p* < 0.001) and at 6 months of benralizumab treatment, with statistically significant differences compared with the results at 3 months (0 [IR:1] vs 0 [IR:0], *p* = 0.003). Similarly, the percentage of corticosteroid-dependent patients decreased by 50% at 6 months (*p* = 0.008) of treatment. In addition, there was also a decrease in the use of ICS (μg/day of budesonide or equivalent), both at 3 months compared with baseline (956 ± 475 vs 802 ± 415, *p* = 0.002) and at 6 months compared with the results obtained at 3 months (802 ± 415 vs 714 ± 356, *p* = 0.020).

**Fig. 4:**
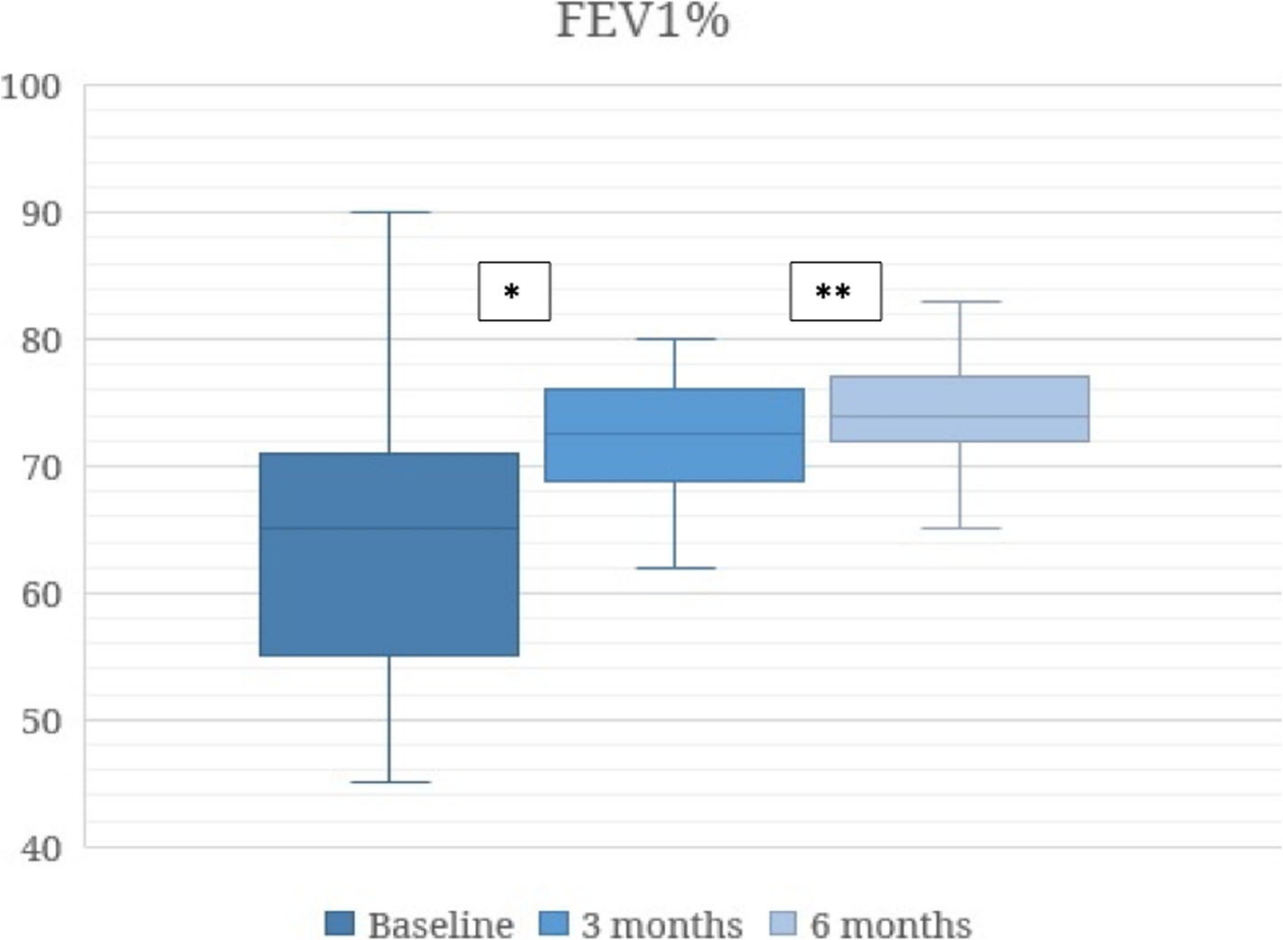
FEV_1_ values in percentage. * *p* < 0.001 (baseline vs 3 months); ** *p* = 0.002 (3 months vs 6 months)

Furthermore, lung function (measured both in mL and as a percentage) also improved. Thus, FEV_1_% improved at 3 months of benralizumab treatment compared with baseline (64.4 ± 9.3 vs 73.1 ± 9.1, *p* < 0.001) and at 6 months of benralizumab treatment compared with 3 months (73.1 ± 9.1 vs 76.1 ± 12, *p* = 0.002), as shown in Fig. 4.

FeNO levels also decreased at 3 months (61.5 ± 23.9 vs 30.7 ± 14.6, *p* < 0.001) and were maintained at 6 months (27.8 ± 14.9).

Blood eosinophil counts were also found to have decreased at 3 months and this decrease was maintained at 6 months.

Of the 42 patients treated with benralizumab, 52.4% had received a biologic agent previously: 15 (35.7%) were receiving omalizumab, 5 (11.9%) mepolizumab, and 2 (4.8%) omalizumab first followed by mepolizumab. Clinical, functional, and laboratory characteristics of biologic treatment-naïve patients (having received no prior biologics) at baseline and at 6 months of benralizumab treatment were compared with those of patients who had previously received biologics. Statistically significant differences were found in IgE levels (216 [288] vs 43.5 [39.5], *p* < 0.001), presence of atopy (52.4% vs 10%, *p* = 0.01), and presence of aspirin-exacerbated respiratory disease (AERD) (27.3% vs 0%, *p* = 0.022), with lower levels of IgE and lower percentages of atopy and AERD in biologic treatment-naïve patients (Table 3). Clinical, functional, and laboratory data presented as the difference between the results at baseline and at 6 months according to prior use of a biologic agent are provided in Table 4. No differences were found in subjective improvement at 3 and 6 months, or in other results at 6 months (measured as the difference between results at 6 months and at baseline) such as lung function (FEV_1_ in mL and %), FeNO, asthma control based on the ACT score, emergency department visits, OCS use, and levels of blood eosinophils and IgE.

**Table 3:** Baseline characteristics according to prior biologic therapy use

Variables	Biologic treatment-naïve patients (***n*** = 20)	Patients with prior biologic therapy (***n*** = 22)	p
Age, years; md (IR)	58 (20.75)	52 (14.75)	0.089
Women, n (%)	16 (80)	17 (77.3)	1
BMI, md (IR)	28 (6.75)	27.5 (8.5)	0.980
Age at diagnosis, years; md (IR)	30 (24)	22.5 (14)	0.079
Atopy, n (%)	2 (10)	12 (54.5)	**0.01**
Corticosteroid-dependent, n (%)	7 (35)	10 (46)	0.708
Nasal polyps, n (%)	3 (15)	9 (41)	0.130
AERD, n (%)	0 (0)	6 (27)	**0.022**
ACT, md (IR)	14 (6)	13.5 (6)	0.430
ED visits in the previous year; md (IR)	5 (2)	3 (6)	0.325
Cycles of OCS in the previous year; md (IR)	7 (2)	5 (7)	0.155
Oral prednisone (or equivalent) dose, mg/day; md (IR)	20 (15)	17.5 (20)	0.579
Post-BD FEV_1_, mL; md (IR)	1420 (835)	1445 (913)	0.980
Post-BD FEV_1_, %; md (IR)	65 (13)	66.5 (16)	0.404
FeNO, ppb; md (IR)	66 (32)	60 (60)	0.351
Blood eosinophil count, cells/μL; md (IR)	755 (315)	660 (205)	0.177
IgE, IU/mL; md (IR)	43.5 (40)	216 (288)	**< 0.001**

Abbreviations: *ACT* Asthma Control Test, *AERD* Aspirin-exacerbated respiratory disease, *BD* Bronchodilator, *BMI* Body mass index, *ED* Emergency department, *FeNO* Fractional exhaled nitric oxide, *FEV*_*1*_ Forced expiratory volume in 1 s, *IR* Interquartile range, *md* Median, *ppb* Parts per billion, *OCS* Oral corticosteroids

**Table 4:** Clinical, functional, and laboratory data presented as the difference between results at baseline and at 6 months according to prior biologic therapy use

Variables	Biologic treatment-naïve patients (n = 20)	Patients with prior biologic therapy (n = 22)	p
Subjective improvement*, n (%)	20 (100)	18 (81.8)	0.487
Corticosteroid-dependent at 6 months, n (%)	4 (20)	4 (18.2)	0.125
ACT*, md (IR)	8 (5)	5.5 (4)	0.196
No. of ED visits at 6 months; md (IR)	1 (5)	2 (10)	1
Post-BD FEV_1_, mL*; md (IR)	330 (350)	238 (375)	0.279
Post-BD FEV_1_, %*; md (IR)	11 (8)	7.5 (15)	0.424
FeNO, ppb*; md (IR)	40 (28)	34 (45)	0.316
Blood eosinophil count, cells/μL*; md (IR)	755 (312)	692 (221)	0.274

*Difference between results at baseline and at 6 months

Abbreviations: *ACT* Asthma Control Test, *BD* Bronchodilator, *ED* Emergency department, *FeNO* Fractional exhaled nitric oxide, *FEV1* Forced expiratory volume in 1 s, *IR* Interquartile range, *md* Median, *ppb* Parts per billion

Likewise, we investigated whether atopy predisposes to a better or poorer response at 6 months of treatment with benralizumab. Baseline clinical, functional, and laboratory characteristics are shown in Table 5 according to patient allergy status. Data presented as the difference between results at baseline and at 6 months according to patient allergy status are provided in Table 6. Differences in clinical parameters (ACT, emergency department visits, and OCS use), functional parameters (FEV_1_ in mL and %), FeNO, and laboratory parameters (blood eosinophils) were investigated. No statistically significant differences were found for any of the parameters.

**Table 5:** Baseline characteristics according to allergy status

Variables	Non-atopic (***n*** = 28)	Atopic (***n*** = 14)	p
Age, years; md (IR)	56 (20)	49.5 (15)	0.153
Women, n (%)	23 (82)	10 (71)	0.451
BMI, md (IR)	28.5 (7.5)	25 (10)	0.131
Age at diagnosis, years; md (IR)	26.5 (23)	24.5 (16)	0.913
Corticosteroid-dependent, n (%)	13 (46)	4 (28)	0.331
Nasal polyps, n (%)	5 (18)	7 (50)	0.067
AERD, n (%)	3 (11)	3 (21)	0.383
ACT, md (IR)	13 (6)	15 (6)	0.730
ED visits in the previous year; md (IR)	5 (3)	3 (3)	0.062
Cycles of OCS in the previous year; md (IR)	7 (3)	4.5 (4)	0.062
Oral prednisone (or equivalent) dose, mg/day; md (IR)	20 (15)	15 (23)	0.412
Post-BD FEV_1_, mL; md (IR)	1520 (910)	1425 (545)	0.947
Post-BD FEV_1_, %; md (IR)	65 (13)	69.5 (12)	0.117
FeNO, ppb; md (IR)	68.5 (32.5)	44.5 (45)	0.098
Blood eosinophil count, cells/μL; md (IR)	713 (297)	695 (235)	0.743
IgE, IU/mL; md (IR)	53 (106)	215 (175)	**< 0.001**

Abbreviations: *ACT* Asthma Control Test, *AERD* Aspirin-exacerbated respiratory disease, *BD* Bronchodilator, *BMI* Body mass index, *ED* Emergency department, *FeNO* Fractional exhaled nitric oxide, *FEV*_*1*_ Forced expiratory volume in 1 s, *IR* Interquartile range, *md* Median, *ppb* Parts per billion, *OCS* Oral corticosteroids

**Table 6:** Clinical, functional, and laboratory data presented as the difference between results at baseline and at 6 months, according to allergy status

Variables	Non-atopic (n = 28)	Atopic (n = 14)	p
Subjective improvement*, n (%)	25 (96)	13 (93)	1
Corticosteroid-dependent at 6 months, n (%)	6 (21.4)	2 (14.3)	0.225
ACT*, md (IR)	8 (3)	5 (4)	0.061
No. of ED visits at 6 months; md (IR)	2 (8)	1 (8)	1
Post-BD FEV_1_, mL*; md (IR)	307 (295)	379 (343)	0.465
Post-BD FEV_1_, %*; md (IR)	11 (7)	14 (15)	0.541
FeNO, ppb*; md (IR)	38 (29)	25 (30)	0.158
Blood eosinophil count, cells/μL*; md (IR)	751 (234)	725 (374)	0.650

*Difference between results at baseline and at 6 months

Abbreviations: *ACT* Asthma Control Test, *BD* Bronchodilator, *ED* Emergency department, *FeNO* Fractional exhaled nitric oxide, *FEV1* Forced expiratory volume in 1 s, *IR* Interquartile range, *md* Median, *ppb* Parts per billion

Among the side effects experienced by 9 patients (21.4%), the most common ones were arthralgias, headaches, and dysthermia. However, all the side effects were mild and did not lead to treatment discontinuation.

## Discussion

Several clinical trials have shown that benralizumab is safe and effective in patients with refractory eosinophilic asthma. However, it is well known that real-life data may differ from data obtained from pivotal studies, as conventional randomised controlled trials emphasise internal validity through standardisation and control, but by design, they reduce external validity and therefore the generalisability of results and conclusions [43]. The present study confirms that in a real-life setting, benralizumab also improves asthma control, reduces emergency department visits and the use of both oral and inhaled corticosteroids, and improves lung function, which is in line with pivotal studies SIROCCO [30], CALIMA [31], and ZONDA [32]. In addition, we have found that outcomes improved with time. In a recent study of 13 corticosteroid-dependent patients in a real-life setting, [44] it was shown that a single dose of benralizumab led to a rapid improvement of asthma control and lung function, to decreased blood eosinophil counts, and to a reduction in the use of OCS. The authors postulate that the rapid therapeutic action observed is a consequence of the fast and effective depletion of eosinophils induced by benralizumab via IL-5R blockade and ADCC-mediated apoptosis of these cells [44]. However, our study suggests that improvement continues with time as results at 6 months are not only better compared with baseline but also compared with the results at 3 months of treatment. Thus, although initial improvement may be rapid and significant, improvement continues during the first 6 months, not only with regard to asthma control but also to lung function, emergency department visits, and use of inhaled and oral corticosteroids.

The major improvement in the ACT score at 3 months of treatment and the improving trend which continued up to the 6 months evaluation are similar to or even better than the results obtained in clinical trials, which have also demonstrated a significant decrease in the asthma control questionnaire (ACQ-6) score [30, 31]. As for the number of emergency department visits in our study, these dropped by 55.8% (*p* < 0.001) at 3 months and continued decreasing until reaching 85.3% (*p* < 0.001) at 6 months, while in the SIROCCO and the CALIMA studies, reductions in exacerbations were 42% per year (*p* < 0.001) [30] and 36% per year (*p* < 0.001) [31], respectively. This greater improvement compared to the pivotal studies could also be associated with the greater disease severity of patients included in our study. The figures in the pivotal studies are clearly lower than those obtained in the present study.

Furthermore, the clear reduction in the use of corticosteroids found in our study, with a reduction of 50% in corticosteroid dependence, is comparable with the data from the ZONDA study [32] in which a 52% decrease in corticosteroid dependence at 28 weeks of treatment was evidenced. The reduction of 61.7% at 3 months and 74.4% at 6 months compared with baseline obtained in the dose of OCS is also comparable with the results from the ZONDA study [32] where a 75% reduction in OCS use at 28 weeks was observed. However, our results are in contrast with those of Pelaia et al. [44] who achieved OCS discontinuation in all the study patients in only four weeks. Given that most of our corticosteroid-dependent patients had been on OCS for years, we decided to perform a corticosteroid tapering similar to the one used in the ZONDA study [32] in order to maintain symptom control and avoid potential side effects resulting from rapid discontinuation. As a result, the highest number of corticosteroid discontinuations was beginning to be observed at 6 months. In fact, we used a tapering-discontinuation protocol similar to the tapering algorithm for OCS published in the PONENTE study [45], a phase 3 clinical trial assessing the efficacy and safety of OCS tapering following benralizumab treatment initiation in adult patients with severe uncontrolled eosinophilic asthma.

As for ICS, a reduction in their use was observed at 3 months of treatment and continued at 6 months, with statistically significant differences compared with the results obtained at 3 months (*p* = 0.020). This is particularly relevant as benralizumab not only seems to reduce the burden of systemic corticosteroids but may also reduce the burden of inhaled agents, an aspect that has not been assessed in other studies.

Lung function improvement (both in mL and percentage) was in line with the clinical trials. The difference between baseline FEV_1_ and FEV_1_ at 6 months was 0.291 L (*p* < 0.001) in our study, whereas the difference with placebo was 0.159 L (*p* = 0.0006) in the SIROCCO study [28] and 0.116 L (*p* = 0.0102) in the CALIMA study [31] in patients with ≥300 eosinophils/μL. Thus, the difference in our study is greater than in the pivotal studies. However, this could be partly explained by the fact that in our study we could not take into account a placebo effect given that, as it was performed in a real-life setting, there was no placebo control group. Other real-life studies such as that of Pelaia et al. [44] have shown improvements in FEV_1_ of about 0.4 L, which would support the hypothesis that lung function improvement is greater in real-life situations. The new information our study provides is that, in addition, lung function continues to improve between 3 months and 6 months of treatment. Therefore, after the improvement that results from the first 3 doses of benralizumab, lung function can continue to improve.

With regard to the effectiveness of benralizumab in patients who had previously received another biologic agent, no differences compared to other patients in the study were found in clinical parameters such as subjective improvement, ACT score, emergency department visits and use of OCS, in lung function (FEV1 in mL or percentage), FeNO, blood eosinophil counts, or in side effects. To our knowledge, this is the first study that shows that benralizumab also improves the condition of patients whose asthma was unresponsive to a biologic treatment targeting the IL5 or IgE pathways. This is especially relevant in the current context of personalised medicine [46] and highlights the need to measure biomarkers to guide treatment decisions [47]. Some studies have shown that patients who do not respond to omalizumab can improve with anti-IL5 therapy. In the case of mepolizumab, *post-hoc* analyses of two pivotal studies [48] evidenced similar reductions in exacerbations and OCS use in asthmatic patients with and without prior omalizumab treatment. In the OSMO study [49] where the main objective was to assess if patients eligible for both biologics, but not optimally controlled with omalizumab, experienced improved asthma control when switched directly to mepolizumab, the results showed that, in patients who were not optimally controlled by omalizumab treatment, switching to mepolizumab improved asthma control and reduced exacerbations. Similarly, a recently published prospective and multicentre study with reslizumab [50] provided evidence that, for patients who do not respond to omalizumab, the switch from omalizumab to reslizumab improves patient control and reduces the use of OCS. Based on our study results showing a clinical improvement in patients who had failed to respond to omalizumab or mepolizumab, we believe that benralizumab is a very good treatment option for patients with severe refractory eosinophilic asthma.

Benralizumab has also been shown to be effective in atopic patients and our results are in agreement with a *post-hoc* analysis [51] of two clinical trials indicating that benralizumab is efficacious in severe uncontrolled eosinophilic asthma regardless of atopy status.

Lastly, in line with published works [33], side effects were mild and well tolerated, with no treatment discontinuations during the first 6 months of treatment.

Our study has some limitations. Because this was a real-life study, there is no placebo control group so placebo effect could not be assessed. Furthermore, even though this study was conducted in a real-life setting with the longest follow-up period to date (6 months), longer follow-up times would be necessary to establish when maximum improvement is reached.

Its strengths, on the other hand, lie in the fact that, to our knowledge, to date this is the real-life study of benralizumab with the greatest number of patients enrolled and the longest follow-up period. In addition, it is a multicentre study that was conducted at two different severe asthma units with a broad experience in the management and treatment of this disease. Finally, this was an independent study with no external funding and without the involvement of any pharmaceutical company.

## Conclusions

Our data show that benralizumab used as add-on therapy in the treatment of refractory eosinophilic asthma is also effective in real life, improving asthma control and lung function, and reducing the number of emergency department visits and the use of oral and inhaled corticosteroids. It is in agreement with pivotal studies and provides a real-life perspective. In addition to demonstrating a rapid initial improvement, our results suggest that patients may continue to improve during the first 6 months of treatment. Furthermore, improvement in the real-life setting appears to be greater than in pivotal studies. Lastly, this study also shows that benralizumab is safe and well tolerated in real life.

## Data Availability

The datasets analysed during the current study are available from the corresponding author upon reasonable request.

## Abbreviations

ACQ-6: Asthma control questionnaire score
ACT: Asthma control test
ADCC: Antibody-dependent cell-mediated cytotoxicity
AERD: Aspirin-exacerbated respiratory disease
BD: Bronchodilator
BMI: Body mass index
FeNO: Fractional exhaled nitric oxide
FEV1: Forced expiratory volume in 1 s
GINA: Global Initiative for Asthma
ICS: Inhaled corticosteroids
IL-5: Interleukin-5
IL-5R: Interleukin-5 receptor
IR: Interquartile range
LABA: Long-acting β2-agonists
OCS: Oral corticosteroids
